# Soy protein isolate film activated by black seed oil nanoemulsion as a novel packaging for shelf‐life extension of bulk bread

**DOI:** 10.1002/fsn3.3864

**Published:** 2023-12-07

**Authors:** Negin Hosseiniyeh, Forogh Mohtarami, Hadi Almasi, Saeedeh Azizi

**Affiliations:** ^1^ Department of Food Science and Technology, Faculty of Agriculture Urmia University Urmia Iran

**Keywords:** mold, *Nigella sativa* oil, Pickering emulsion, whey protein isolate, white bread

## Abstract

This paper investigates the addition of lecithin‐emulsified black seed oil (BSO) nanoemulsions (LNEO) and whey protein isolate‐stabilized Pickering emulsions (WPEO) to soy protein isolate (SPI)‐based films and their effect on improving the shelf life of bread slices. The half‐life of antioxidant activity, water vapor permeability, biodegradability, density, color difference, and film thickness significantly increased (*p* < .05) when BSO was added. However, the incorporation of BSO significantly reduced the solubility, tensile strength, strain to break (except for WPEO), and transparency (*p* < .05) of the samples. The interaction between SPI film and BSO‐loaded nanocarriers, as well as the morphological properties of films, was evaluated using FT‐IR and FE‐SEM. SPI‐based films containing LNEO‐5% and WPEO‐5% were selected based on their mechanical and barrier properties. The effect of films on the shelf life of bread slices was investigated for 17 days of storage. LNEO samples obtained the most acceptable results in the bread in terms of sensory evaluation and color properties. According to the results, bread slices packed in SPI film containing LNEO‐5% showed no signs of mold growth until the 17th day of storage, whereas the sample packed in a low‐density polyethylene bag began to corrupt on the 6th day. This study highlights the potential of BSO‐loaded SPI films as a novel active packaging for the bakery industry.

## Graphical abstract

Since bread is one of the most consumed products in the world, with a limited shelf life (2–4 days at room temperature), using new approaches to prevent the growth of microorganisms such as active packaging with natural and healthy antimicrobial compounds is the main challenge. *Nigella sativa* oil contains thymoquinone, dithymoquinone (nigellone), thymohydroquinone, and thymol as main active (black seed) constituents which could be used as a natural preservative against mold growth. Based on the problems associated with the shelf life of bread and increasing the demands of consumers to decrease the usage of chemical preservatives, this study aimed to investigate the black seed oil as nanoemulsions (NE) and Pickering emulsions (PE) in soy protein isolate (SPI)‐based films and their effect on improving white bread slices' shelf life. According to the results, white bread slices packed in LEO‐5% film did not show any mold growth until the 17th day of storage, whereas control samples were spoiled on the 6th day.

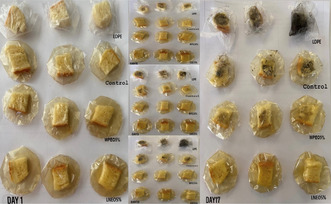

## 1 INTRODUCTION

Bread is one of the most consumed products in the world. It has a short shelf life of 2–4 days at room temperature due to its high moisture content, microbial spoilage, and textural changes (Qian et al., 2021). Generally, *Aspergillus*, *Rhizopus*, *Eurotium*, *Endomyces*, *Penicilliumroqueforti*, *Penicilliumpolonicum*, and *Penicillium* are the primary fungi associated with the deterioration of bakery products, which create an unpleasant taste and endanger the health of consumers by producing mycotoxins (Jafarzadeh et al., 2022). *Penicillium* is responsible for the spoilage of more than 90% of wheat products (Schettino et al., 2020). Some preservatives, such as sodium propionate, calcium acetate, and sodium sorbate, are considered safe in the bakery industry, but their use is unsatisfactory due to their resistance to fungal strains, low solubility, and toxicity. Consumers are reluctant to include chemical preservatives in their food, especially bread, which they consume daily, so using new approaches to prevent the growth of microorganisms such as active packaging loaded with natural and healthy antimicrobial compounds can be effective (Gavahian et al., 2020; Qian et al., 2021). Today, biodegradable films containing antioxidant and natural antimicrobial materials have received much attention. In active packaging, the antimicrobial agent is loaded into the packaging material instead of being added directly to the food. There are some reports on the application of active packaging for the shelf‐life extension of bakery products. For example, poly(3‐hydroxybutyrate‐co‐4‐hydroxybutyrate) films incorporated with thyme essential oil (EO) have been used for packaging white bread and were able to extend the shelf life up to 5 days (Sharma et al., 2022). Heras‐Mozos et al. (2019) developed an active film based on polyethylene and zein containing garlic extract for packaging the sliced pan loaf. Their results exhibited that by using active film, the bread was free of mold for 30 days. It has been demonstrated that active packaging based on ethylene‐vinyl alcohol containing geraniol prolongs the shelf life of bread slices by more than 3 weeks (Cheng, 2018).

Among the biopolymeric film‐forming materials, soy protein isolate (SPI) has received much attention as one of the most attractive alternatives to synthetic polymers due to its high mechanical strength, good transparency, biocompatibility, and biodegradability (Tang et al., 2019; Xiao et al., 2021). The SPI has been used for the fabrication of active films by incorporation of various biologically active substances such as oils (Binsi et al., 2013; Bohórquez‐Ayala et al., 2021; Chen et al., 2020; Giannakas et al., 2021; Ma et al., 2015; Sultan et al., 2022), plant extracts (Almasi et al., 2016; Aziz & Almasi, 2018; Haghju et al., 2016; Hiremani et al., 2022; Homayounpour et al., 2021), and EOs (Alinaqi et al., 2021; Gasti et al., 2022; Sadadekar et al., 2022; Sharma et al., 2022; Zhang, He, et al., 2022; Zhang, Jiang, et al., 2022).

*Nigella sativa*, also known as black seed, is an annual flowering plant of the Ranunculaceae family. The use of black seeds and their oil has been recommended for rheumatoid arthritis, asthma, inflammatory diseases, diabetes, and digestive diseases. *N. sativa* seeds contain unsaturated fatty acids (26%–38%), proteins, alkaloids, saponins (melanin), and EO (0.4%–2.5%; Makouie et al., 2021). Thymoquinone, dithymoquinone (nigellone), thymohydroquinone, and thymol are considered the main active constituents of black seed oil (BSO). The predominant alkaloids that have been removed from *N. sativa* seeds are nigellicine, nigellidine (indazoles), nigellimine, and nigellimine N‐oxide (isoquinolines). Furthermore, both nigellimine and thymoquinone from *N. sativa* might be considered as potential medicinally bioactive components to treat COVID‐19 patients (Rahman, 2020). The antimicrobial and antioxidant potential of BSO makes it a candidate for use as an active agent in food packaging. However, directly adding bioactive compounds to films restricts the effectiveness of preservatives due to partial inactivation of the bioactive compounds because of the interaction with food components, which can also adversely affect the sensory properties of the product. Moreover, uncontrolled rapid migration of active compounds and their decomposition during food processing, poor miscibility, phase separation during the film‐forming process, and instability of bioactive compounds against environmental conditions (oxygen, light, temperature, humidity, and pH) are other limitations associated with directly adding bioactive compounds to film matrix (Hemmatkhah et al., 2020; Ribeiro‐Santos et al., 2018). All these factors can impact the performance of the active film, and consequently reduce the activity of the film during the shelf life of food (Almasi et al., 2020).

One approach to overcome these limitations is the use of active compounds in the encapsulated form. Encapsulation is the process of trapping active substances inside another wall material. The most appropriate nanoscale carriers for food applications are lipid‐based nanocarriers and biopolymer‐based nano‐ or microcapsules. Nanoemulsion (NE) as an oil‐in‐water emulsion with a lipid droplet size of 10–100 nm is one of the lipid‐based nanocarriers. Higher transparency, less tendency to phase separation, enhanced physicochemical properties, improved controlled release and stability, and improved biological activity are the main advantages of NE formation. The incorporation of EOs and other active compounds within the NEs form for activation of biopolymer‐based active films and coatings has been reported (Ansarian et al., 2022; Shen et al., 2021; Zhang et al., 2021). Lecithin has been used in many NE‐loaded films due to its strong emulsifying properties (Elshamy et al., 2021; Kaur & Singh, 2021; Liao et al., 2021). Another sustainable delivery system is biopolymer‐stabilized microcapsules called Pickering emulsion (PE). Unlike low‐molecular‐weight surfactants, when solid particles are adsorbed, a thicker interface layer can be formed at the oil–water phase interface, leading to advantages such as higher coalescence stability, stronger protection of the encapsulated compound, higher loading capacity, and lower release rate (Liu et al., 2022; Zhang, He, et al., 2022; Zhao et al., 2022). Shen et al. (2021) prepared clove EO‐loaded NE‐ and PE‐activated pullulan‐gelatin‐based film. Particularly, films with PE showed higher mechanical and water barrier properties and provided a slower release profile compared to NE. Proteins such as whey protein isolate (WPI) are the most suitable biopolymers for encapsulating EOs in the form of PE due to their hydrophobic and hydrophilic amino acids. Recently, PE and NE capsules with marjoram EO (MEO) as oil phase and WPI and inulin as stabilizers were prepared and added to the pectin film. The addition of MEO‐loaded PE in the pectin film increased the tensile strength and reduced the water vapor permeability, while the MEO‐loaded NE pectin film showed higher antibacterial activity (Almasi et al., 2020).

To the best of our knowledge, there is no report on the preparation and characterization of BSO nanocapsules. Moreover, the effect of BSO‐loaded active films on the shelf‐life extension of bread or other food products has not been investigated. This study aimed to produce an active packaging film based on SPI containing BSO encapsulated NE and PE and to investigate its physicochemical and mechanical properties as well as the application of the fabricated film for packaging and shelf‐life extension of sliced bulk bread.

## 2 MATERIALS AND METHODS

### 2.1 Materials

The pure and cold‐pressed *N. sativa* oil (also called BSO) was purchased from a local market, in Urmia, Iran. The oil was packed in a dark bottle and stored in a cool and dark place until analysis. The used chemicals including lecithin, SPI (95% protein), WPI (90% protein), glycerol, and methanol 99.8% were obtained from Merck (Darmstadt, Germany). All chemical reagents like 2‐diphenyl‐1‐picrylhydrazyl (DPPH) were provided by Sigma‐Aldrich (Gillingham, Dorset, UK). Distilled water was used in all experiments.

### 2.2 Preparation of NE‐ and PE‐stabilized BSO

The method of Noori et al. (2018) was used for the preparation of BSO‐loaded NE (LNEO). First BSO (1 wt%) and lecithin (30 wt% of BSO) were added to distilled water gradually and continuously while agitating at 3000 rpm to obtain an initial coarse emulsion which was O/W type. Then, ultrasonic emulsification through a probe sonicator (OPTIMA, XL100K, Germany) operating at 200 W and 20 kHz was employed to develop NE. The probe of the device with a 15 mm diameter was dipped into coarse emulsion at a depth of 25 mm and did so for 15 min at room temperature. The WPI‐stabilized PE was prepared by the method of Almasi et al. (2020). The biopolymer suspension containing WPI (4 wt%) in distilled water was prepared the day before encapsulation to ensure complete saturation of biopolymer molecules and kept for 24 h at room temperature. Afterward, BSO at an amount of 1 g was slowly added to the biopolymer dispersion (core‐coating ratio at 1:4) by stirring at 5000 rpm to prepare the pre‐emulsion. The probe sonicator with the same conditions mentioned above was used for encapsulation of BSO by WPI (WPEO). These samples were freeze‐dried for 48 h at −40°C and vacuum of 0.007 atm before evaluating with FE‐SEM analysis.

### 2.3 Characterization of NE‐ and PE‐stabilized BSO

The volumetric mean diameter (D_43_), zeta potential, and polydispersity index (PDI) of LNEO and WPEO were determined using the method of Almasi et al. (2020), according to dynamic light scattering (DLS) method using Zetasizer (Zetasizer Nano‐ZS, Malvern, UK). All measurements were performed at 25°C in three replicates. Structural interactions of NE‐ and PE‐stabilized BSO samples were analyzed using Fourier Transform Infrared (FTIR) analysis, by FTIR spectroscopy (SHIMADZU IRPrestige/FT‐IR‐8000, Japan) in the range of 4000–400 cm^−1^ at a 4 cm^−1^ resolution. The KBr pellet tablet method was used to prepare the sample. Fluorescence microscopy (Bh2‐RFCA, Olympus, Japan) and field emission scanning electron microscopy (FE‐SEM; Zeiss, Sigma, Germany) were employed to observe the shape and morphology of LEO and WEO samples, respectively. The accelerator voltage was from 10 to 20 kV.

The encapsulation efficiency (EE) of LNEO and WPEO was measured by putting 1 mL of LNEO and WPEO into an Amicon® filter (molecular weight cutoff 100 kDa, Millipore, UK) after dilution with distilled water. Separation of unencapsulated BSO from capsules was performed by centrifuging (Universal 320 centrifuge, Hettich, Germany) the samples at 2000 rpm for 10 min. The unloaded oil concentration was then determined by UV–Vis spectroscopy (Unico, S 2100 SUV, Dayton, Japan) and calibration curve. BSO had a maximum absorption at 244 nm (*λ*_max_), and measurements were made at this wavelength. Finally, EE was calculated by Equation 1 (Almasi et al., 2020):

(1) $$\mathrm{EE}\;\left(\%\right)=\frac{\text{Total}\;\mathrm{BSO}\;\text{used in emulsion}\;\left(\mathrm{mg}\right)-\text{Free}\;\mathrm{BSO}\;\text{content}\;\left(\mathrm{mg}\right)}{\text{Total}\;\mathrm{BSO}\;\text{used in emulsion}\;\left(\mathrm{mg}\right)}\times 100$$

### 2.4 Film preparation

The SPI films were prepared using the method outlined by Ghadetaj et al. (2018) with some modifications. To prepare the BSO‐loaded films, 4 g of SPI was dispersed in 100 mL of distilled water at room temperature. The mixture was stirred with a magnetic stirrer for 1 h. Subsequently, the pH of the solution was adjusted to 10 with NaOH 0.1 N and heated for 45 min at 70°C for denaturation of WPI. Glysrol (35% w/w of SPI) was then added as a plasticizer to the cooled solution. Afterward, free BSO (FO) and emulsion forms of BSO (LNEO and WPEO) were added based on EE to the SPI solution to maintain the concentrations of BSO at constant levels (5 and 10% w/w of SPI). For complete mixing, agitation was done for 15 min with a magnetic stirrer. A pure SPI film without BSO was prepared as a control sample under the same conditions. Approximately, 25 mL of each film‐forming solution was poured into plastic Petri dishes with a diameter of 10 cm and dried at 27 ± 5°C (ambient temperature) for 48 h.

### 2.5 Characterization of active SPI films

#### 2.5.1 Film thickness and solubility

The thickness of the films was measured by a digital micrometer (Fowler, USA) with an accuracy of 0.001 mm at 10 random points of each film sample. The average thickness of different points of films was used to calculate the mechanical properties and water vapor permeability (Almasi et al., 2020). Film solubility was assessed based on the method of Daei et al. (2022). Weighed film samples (2 × 2 cm^2^) were immersed in 300 mL of distilled water and stirred at 25°C for 3 h on a magnetic stirrer. Excess water from the films was removed using a paper filter, and afterward, the samples were dried in a forced‐air oven at 105°C for 24 h. The percentage of solubility was calculated according to the following equation:

(2) $$\text{Solubility}\;\left(\%\right)=\frac{m0-m1}{m0}\times 100$$

where m_0_ is the initial weight of the film (g) and m_1_ is the final weight of the dried film (g).

#### 2.5.2 Water vapor permeability

Water vapor permeability (WVP) test was conducted according to Daei et al. (2022). The films were cut into disc shapes and put within the cap of vials containing 3 g calcium sulfate (RH = 0%). Following the initial weighing, the vials were placed in a desiccator that contained potassium sulfate (RH = 97%). The quantity of water vapor absorbed by the calcium sulfate from the film was subsequently determined by measuring the weight increase of the vials. The vials were weighed at regular intervals for 72 h. The water vapor transmission rate (WVTR) (g m^−2^ h^−1^) was calculated from the slope obtained from the regression analysis of moisture weight gain (Δ*w*) transferred from a film area (*A*) over a specific time (Δ*t*). The WVP (g.mm m^−2^ h^−1^ Pa^−1^) was determined using the following equations:

(3) $$\text{WVTR}=\frac{∆w}{A∆t}$$

(4) $$\mathrm{WVP}=\frac{\text{WVTR}\times X}{\Delta P}$$

where *A* is the film area (m^2^), *X* is the film thickness (mm), and ∆*P* is equal to the difference of water vapor pressure between the inner and outer surface of the film in vials (∆*P* = 3169 Pa). All the measurements were carried out in three replicates.

#### 2.5.3 Color and opacity

The color parameters, namely L* (lightness/brightness), a* (redness/greenness), and b* (yellowness/blueness) of film samples, were investigated according to the method presented by Almasi et al. (2020). Briefly, the film samples and RAL standard color sheets were placed inside the standard box and imaged using a digital camera (Canon Power Shot SX720 HS, Japan). The L*, a*, and b* factors of film samples and RAL standard color sheets were proved by using Adobe Photoshop software. Then, the calibration curves were obtained by drawing the actual L*, a*, and b* values of the standard sheets against the shown values by the software. Finally, the L*, a*, and b* values of film samples were calculated via the replacement of software data in the equations of calibration curves. The obtained L*, a*, and b* values were used for the calculation of the total color difference (Δ*E*) of film samples (Almasi et al., 2020):

(5) $$\Delta E=\sqrt{{\left(\Delta L\right)}^{*2}+{\left(\Delta a\right)}^{*2}+{\left(\Delta b\right)}^{*2}}$$

where ΔL*, Δa*, and Δb* are the differences between the color of a standard white color plate (L* = 97.41, a* = − 5.09, and b* = 7.13) and film samples.

The transparency is the absorption ratio of the sample at 600 nm with the specified thickness of the films (mm). Opacity was measured over the standard white tile and black glass in the visible–ultraviolet light range (360–800 nm). All the measurements were carried out in three replicates.

#### 2.5.4 Mechanical properties

The ultimate tensile strength (UTS) and strain to break (STB) of the film samples were determined by a Texture Analyzer (TA.XTplus, Stable Micro System) equipped with a 100 N load cell. After conditioning at RH = 55% for 24 h, the film samples were cut into dumbbell shapes (8 × 0.5 cm^2^) and mounted in two grips at 30 mm apart. The crosshead speed was set to 0.83 mm min^−1^.

(6) $$\mathrm{UTS}=\frac{F_{\max}}{A}$$

(7) $$\mathrm{STB}=\frac{L_{\max}}{L_{0}}\times 100$$

where *A* is the cross‐sectional area of the film (m^2^), *F*_max_ is the maximum force at the breaking point (N), *L*_max_ is the elongation of the film at the breaking point (m), and *L*_0_ is the initial length of the film sample (m). Density was calculated by dividing the mass of the sample by the volume of the films. Film samples measuring 2 cm × 2 cm were weighed, and the volume of the films was determined using their dimensions (area and thickness).

#### 2.5.5 Antioxidant activity and biodegradability

The free radical scavenging activity of the films was determined following the method described by Jahed et al. (2017), based on the red or purple decolorization of DPPH as a reactant. In brief, 25 mg of each film sample was dissolved in 4 mL of distilled water with continuous stirring. Then, 2 mL of film extract solution was mixed with 0.2 mL of DPPH solution (1 mM) in methanol. The resulting solution was stirred for 5 min and then kept in a dark room at room temperature for 1 h. The reduction in absorbance at 517 nm was determined with a UV–Vis spectrophotometer. Finally, the antioxidant activity was measured as the percentage of DPPH free radical scavenging activity using Equation 8 over time. The half‐life of antioxidant activity then was calculated for a first‐order kinetic equation. The biodegradability of the films was also measured by Nouraddini et al. (2018) method. The film samples were cut to 2 cm × 2 cm and were buried on a metal net at a depth of 2 cm. The samples were stored at 25°C under certain aerobic conditions and humidity. The film samples were then excavated after 14 days.

(8) $$\text{Free radical scavenging activity}\%=\frac{{\mathrm{Abs}}_{\text{control}}-{\mathrm{Abs}}_{\text{sample}}}{{\mathrm{Abs}}_{\text{control}}}\times 100$$

#### 2.5.6 Field emission scanning electron microscopy

The surface morphology of the microcapsule solutions and SPI films was studied by Field emission scanning electron microscopy (FE‐SEM; Zeiss, Sigma, Germany) after the gold coating of samples (DST1, Nanostructured Coating Co., Tehran, Iran). The accelerating voltage was from 10 to 20 kV.

#### 2.5.7 FTIR spectroscopy

FTIR spectroscopy (SHIMADZU IRPrestige/FT‐IR‐8000, Japan) was used to observe the structure of the film and nanocarrier solution samples. The spectra were collected over the wave number range of 500–4000 cm^−1^ with a resolution of 4 cm^−1^. The KBr pellet method was used for sample preparation.

### 2.6 Bulk bread preparation and packaging by active films

The preservative‐free bulk bread samples were baked on a pilot scale in the laboratory. The bread was prepared by mixing 100 g flour with 1.5% salt, 1.5% yeast, 1% sugar, and 50% water (based on flour weight). After mixing the ingredients, the dough was allowed to rest for 90 min at 30°C. It was then divided into 30 g portions, rounded, and left for final proofing at 30°C for 30 min. Finally, the dough was baked for 15 min at 220°C in an electric oven (Alton, V402 model, Iran) and cooled at room temperature (25 ± 1°C) for 1 h. Afterward, 2 cm × 2 cm samples were cut from the loaves and completely packed using pure SPI (without BSO), as well as SPI‐based films containing LNEO‐5% and WPEO‐5%. These were compared to slices of bread packed in low‐density polyethylene (LDPE). The samples were stored at room temperature over storage time. The selection of films was based on their mechanical properties and optimal WVP.

### 2.7 Sliced wheat bread quality testing

#### 2.7.1 Microbiological analysis

Bread slices packed in BSO‐loaded SPI films (WPEO5%, LNEO5%), pure SPI film, and LDPE package (as synthetic packaging) were assessed over 17 days by visual inspection of the mold growth. Microbial shelf life was observed from the day of packaging to the day of microbial observation. Slices of bread were considered unacceptable if one of the samples exhibited visible growth of molds.

#### 2.7.2 Evaluation of organoleptic properties

The organoleptic evaluation was performed by 15 semi‐trained panelists consisting of Food Science and Technology College staff and students (Urmia University, Iran) based on the 5‐point hedonic test. Acceptability of bread's appearance, odor, taste, flavor, texture, and overall preference were evaluated on 1, 3, 6, and 9th day after baking. In this method, 5 indicates the most liked and 1 indicates the most disliked of each attribute.

#### 2.7.3 Color properties of bread crust

Bread crust analysis was performed over 17 days of storage by determining optical properties (L*, a*, and b*) by a handheld Minolta colorimeter, and data were configured using SpectraMagic NX software (Minolta CM‐400, Konica Minolta Sensing Americas Inc., Ramsey, NJ, USA).

### 2.8 Statistical analysis

All measurements were conducted in triplicate. The analysis of variance (ANOVA) was applied to the data, and significant differences between the mean values were compared using Duncan's test. One‐way ANOVA was employed to investigate the effect of the film samples on their properties. Meanwhile, two‐way ANOVA was used to assess the effects of storage time and packaging type on the color values and sensory properties of the packed bread slices. All data are presented as mean ± standard deviation. SPSS 23.0.0 statistical software (SPSS Inc., Chicago, IL, USA) was used for data analysis.

## 3 RESULTS AND DISCUSSION

### 3.1 Characterization of encapsulates

#### 3.1.1 Droplet size, PDI, and EE

**FIGURE 1:**
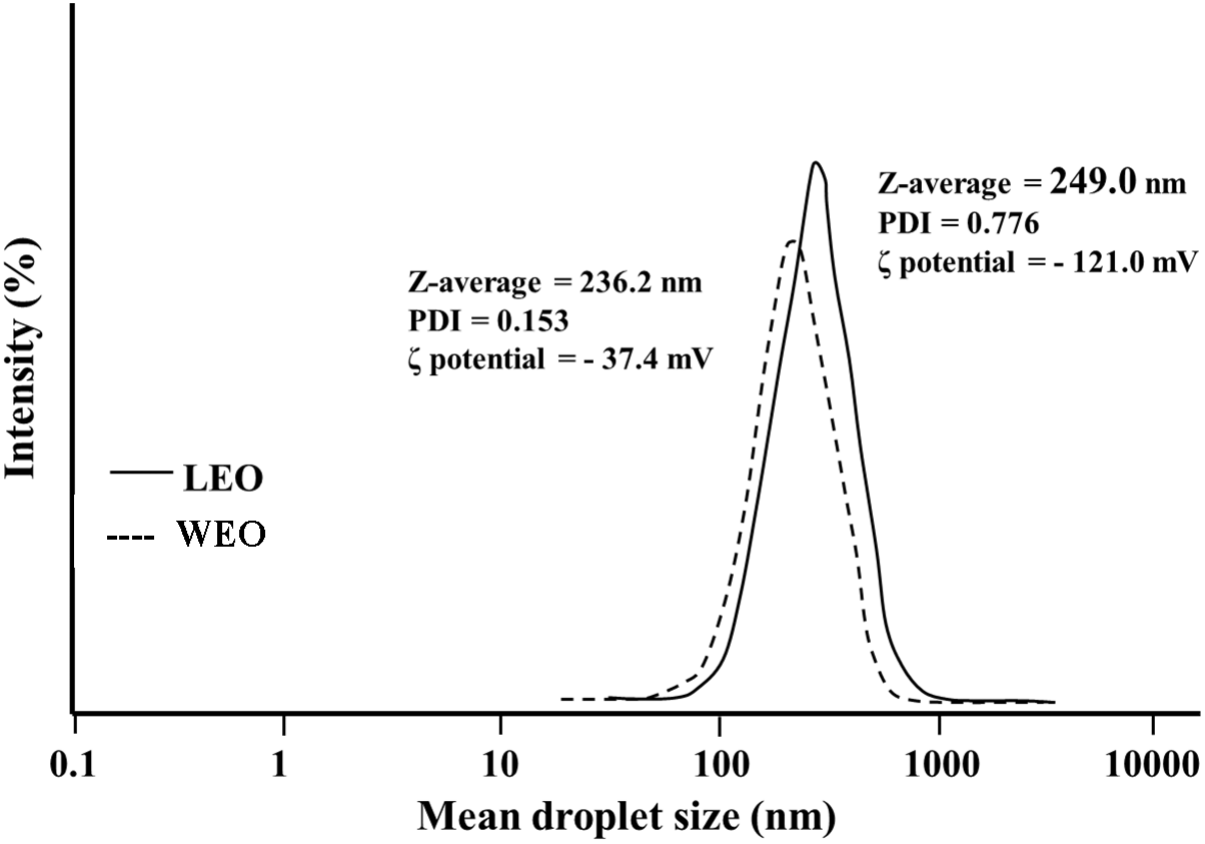
The droplet size, PDI, and zeta potential of black seed oil‐loaded nanoemulsion in lecithin (LNEO), and Pickering emulsion in whey protein isolate (WPEO). PDI, polydispersity index.

The droplet size and PDI have an important effect on microcapsules' stability, optical properties, and release characteristics (Almasi et al., 2020). Figure 1 shows the results of the droplet size, PDI, and zeta potential of LNEO and WPEO. As shown, LNEO exhibited a smaller droplet size of 236.2 nm. When WPI was used as the wall material, the particle size increased to 249 nm. This increase can be attributed to the surface coverage of BSO droplets with macromolecular WPI, which has a higher molecular weight compared to small surfactants (Almasi et al., 2020). Almasi et al. (2020) reported similar results for NE‐ and PE‐stabilized marjoram essential oil encapsulates. However, the size of WPEO nanocarriers is smaller than those reported for other PEs used in the film formulations. For example, the sizes of rosemary EO‐loaded PE (Fasihi et al., 2017) and thymol‐loaded PE (Zhu et al., 2018) were 15 and 10 μm, respectively. Sultan et al. (2022) also reported that the optimum particle size of nano‐sized droplets of coconut oil emulsified by PE using chitosan/Arabic gum nanoparticles was 246.4 nm. In contrast, Fernandes et al. (2017) used the spray‐drying method to encapsulate ginger EO using a combination of WPI and inulin. The larger particle size of the obtained microcapsules (850–1100 nm) in comparison to the current research shows that the ultrasonic method for preparing the PE is more suitable than the spray‐drying method. Makouie et al. (2021) reported that the particle size of microencapsulated *N. sativa* seed oil by freeze‐drying was 749.4 nm.

Small PDI values indicate a narrow size distribution and a low tendency of the capsules to aggregate (Almasi et al., 2020). According to Figure 1, the PDI indexes for LNEO and WPEO samples were 0.153 and 0.776, respectively. This narrow pattern of size distribution confirms the uniformity of the NE and PE droplets organized through the ultrasonication method, making it suitable for efficient use in film formulation (Almasi et al., 2020). The larger PDI values of WPEO could be attributed to the higher molecular weight of WPI in comparison to lecithin, which makes it difficult to tightly pack on the interface.

The zeta potential, which represents the net surface charge of droplets, has an important effect on the stability of the colloidal system (Ghadetaj et al., 2018). The increase in the surface charge of particles enhances the stability of emulsion against creaming and/or flocculation phenomena. This parameter is strongly affected by the type of wall material. The value of zeta potential for LNEO was −37.4 (Figure 1). This relatively low surface charge is attributed to the use of lecithin, which is a zwitterionic surfactant. Similar results have been reported for ginger EO (Noori et al., 2018) and MEO (Almasi et al., 2020) nanoemulsions stabilized by Tween 80. The WPEO nanocarriers exhibited the highest negative zeta potential (−121.0 mV), which is attributed to the negative charge of WPI at pH values above their isoelectric pH (pI). Consequently, the preparation of the microcapsules at neutral pH results in a net negative charge on the capsules stabilized by the WPI. The obtained values are consistent with the results reported by Hemmatkhah et al. (2020) regarding the encapsulation of cumin EO in the form of NE and PE. The results of this study show that biopolymer‐stabilized PE microcapsules produce larger particles with sufficient electrostatic repulsion and higher thermodynamic stability. Also, considering the droplets' PDI and Zeta potential, it is concluded that the uniformity of the droplets is high enough for efficient use in film formulation.

The EE of WEO samples was 61.03 ± 1.78%, while the EE for LNEO microcapsules was about 84.28 ± 1.65%. This higher EE could be related to the lower molecular weight of lecithin, and thus, better surface activity and higher mobility of this compound compared to high‐molecular‐weight biopolymers. The EE of WPEO and LNEO is consistent with the report of Talón et al. (2019), who reported that the efficiency of lecithin‐encapsulated eugenol is higher than that of whey protein‐coated eugenol.

#### 3.1.2 Morphological properties

**FIGURE 2:**
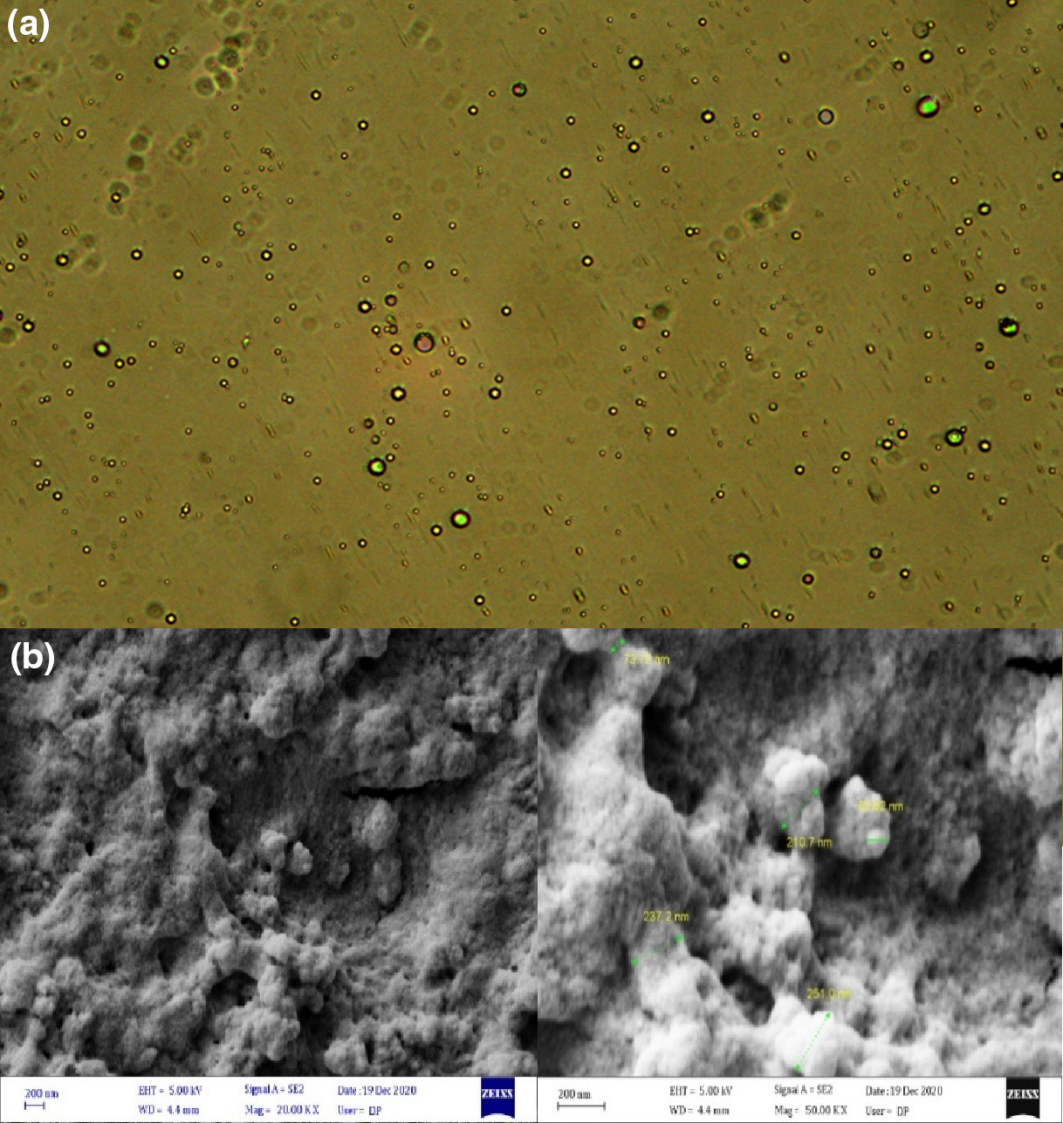
(a) The fluorescence microscope image of black seed oil‐loaded nanoemulsion in lecithin (LNEO) samples. (b) The FE‐SEM images of freeze‐dried Pickering emulsion in whey protein isolate (WPEO)‐stabilized microcapsule powder.

A fluorescence microscope and FE‐SEM microscope were used to evaluate the morphology of LNEO and WPEO, respectively. Figure 2a shows the fluorescence microscopy image of LNEO samples. Spherical droplets with a relatively uniform size distribution are evident. The range of droplet size distribution in the microscopic image, calculated with ImagJ software, was between 205 and 230 nm, confirming the DLS results. Figure 2b shows the FE‐SEM images of freeze‐dried WPEO‐stabilized microcapsule powder. The morphological study shows that all particles encapsulated with biopolymers exhibit a relatively regular spherical shape and a relatively uniform particle size distribution. The average particle diameter of the PE powders was measured at 240 nm, which is consistent with the results of DLS, revealing the successfulness of the ultrasonic method in PE formation.

### 3.2 Characterization of active films

#### 3.2.1 Thickness and water solubility

The thickness of films had a significant effect on mechanical properties and WVP. Results showed that the thickness of films based on SPI varied from 0.166 to 0.226 mm (Table 1). The Control films and those containing FO10% showed the lowest and highest thickness compared to other films (*p* < .05). The thickness of the films increased significantly (*p* < .05) with increasing the amount of BSO as free, PE (WPEO), or NE (LNEO). Similar results were reported by Ma et al. (2015), Mohammadi et al. (2022), and Sharma et al. (2022), who found that an increase in NE and PE content led to greater film thickness. This may be related to the fact that the incorporation of FO, LNEO, and WPEO in the film enhances the compactness of the film matrix, resulting in increased film density (Mohammadi et al., 2022).

**TABLE 1:** Physical and mechanical properties of film samples.

Film sample	Thickness (mm)	Water solubility (%)	Density (g/cm^3^)	WVP (g.mm/m^2^.h.pa)	Strain to break (%)	Ultimate tensile strength (N m^−2^)	Half‐life of antioxidant activity (day)	L*	a*	b*	∆*E*
Control	0.166 ± 0.002^f^	25.10 ± 0.66^a^	0.137 ± 0.0013^b^	0.0055 ± 0.00005^f^	33.56 ± 0.07^c^	7.60 ± 0.10^a^	13.31 ± 0.015^f^	90.55 ± 0.04^a^	−4.16 ± 0.020^a^	4.050 ± 0.010^g^	7.575 ± 0.038^g^
FO5%	0.213 ± 0.005^b^	23.15 ± 0.67^b^	0.103 ± 0.0019^g^	0.0071 ± 0.00010^b^	11.67 ± 0.15^g^	5.39 ± 0.16^e^	18.16 ± 0.005^e^	85.14 ± 0.01^c^	−4.37 ± 0.015^d^	4.526 ± 0.015^b^	12.562 ± 0.018^e^
FO10%	0.226 ± 0.004^a^	20.19 ± 0.45^c^	0.116 ± 0.0018^f^	0.0076 ± 0.00002^a^	18.27 ± 0.08^f^	2.95 ± 0.05^g^	17.94 ± 0.045^e^	82.88 ± 0.01^f^	−4.43 ± 0.010^e^	4.723 ± 0.020^a^	14.744 ± 0.013^b^
LNEO5%	0.175 ± 0.002^de^	23.53 ± 0.34^b^	0.124 ± 0.0010^d^	0.0057 ± 0.00005^e^	29.74 ± 0.15^e^	7.43 ± 0.15^b^	45.09 ± 0.083^c^	84.62 ± 0.02^d^	−4.21 ± 0.015^b^	4.173 ± 0.015^e^	13.158 ± 0.023^d^
LNEO10%	0.189 ± 0.001^c^	23.86 ± 0.69^ab^	0.119 ± 0.0038^e^	0.0061 ± 0.00004^d^	31.14 ± 0.12^d^	4.36 ± 0.15^f^	37.23 ± 0.251^d^	81.98 ± 0.01^g^	−4.35 ± 0.010^d^	4.260 ± 0.010^d^	15.713 ± 0.011^a^
WPEO5%	0.172 ± 0.001^e^	25.11 ± 0.70^a^	0.133 ± 0.0007^c^	0.0066 ± 0.00011^c^	53.91 ± 0.10^b^	7.01 ± 0.07^c^	92.30 ± 0.300^b^	86.66 ± 0.02^b^	−4.23 ± 0.010^b^	4.130 ± 0.020^f^	11.196 ± 0.025^f^
WPEO10%	0.180 ± 0.002^d^	24.38 ± 0.92^ab^	0.144 ± 0.0010^a^	0.0077 ± 0.00006^a^	54.56 ± 0.11^a^	6.15 ± 0.05^d^	94.30 ± 0.360^a^	84.22 ± 0.02^e^	−4.30 ± 0.010^c^	4.380 ± 0.010^c^	13.498 ± 0.022^c^

*Note*: Data are the means of three replicates. Means with different letters within a column indicate significant differences (*p* < 0.05). * used to show the color parameters.

Abbreviations: Control, pure soy protein isolate; FO, free oil; LNEO, black seed oil‐loaded nanoemulsion in lecithin; WPEO, Pickering emulsion in whey protein isolate.

The solubility of the film is an important parameter in different applications, which depends on various factors such as high‐density intermolecular interactions, and in particular the presence of intermolecular covalent bonds of reactive groups (Fakhouri et al., 2018). Additionally, the solubility of SPI film depends on the type, concentration, and hydrophilicity of additives. It is expected that the film's solubility will decrease with the addition of hydrophobic compounds. According to the results presented in Table 1, the addition of BSO led to a decrease in the solubility of the films. The control film (25.10 ± 0.66%) and FO10% film (20.19 ± 0.4%) exhibited the highest and lowest solubility, respectively, in comparison to the other films (*p* < .05). These results were in agreement with the findings of Syarifuddin et al. (2020) who found that the solubility of carrageenan and gelatin‐based films decreased significantly with increasing canola oil content. The presence of epoxidized sesame oil (Bohórquez‐Ayala et al., 2021) and cinnamon EO (Mohammadi et al., 2022) decreased the water solubility of the films, which could be ascribed to the increased hydrophobic component of films. The incorporation of oil into the films increased the superficial hydrophobicity of the films which can reduce film solubility and improve the water resistance of packaging materials to help in extension of the shelf life of moisture susceptible foods.

#### 3.2.2 Water vapor permeability

The results of water vapor permeability (WVP) are presented in Table 1. In general, the incorporation of BSO leads to an increase in the WVP values of the films, from 0.0055 to 0.0077 g.mm m^−2^.h.Pa (*p* < .05). Notably, the lowest WVP was related to pure SPI films (control), followed by LNEO5% ones. By adding and increasing the BSO concentration, the WVP of the films increased significantly (*p* < .05). The maximum WVP value was observed in WPEO10% and FO10% films. This relationship suggests that larger oil droplet sizes correspond to higher WVP values. This phenomenon may be attributed to the disruptions in the polymer network caused by lipid droplets, resulting in a reduction in film cohesion and an increase in transport phenomena through the film. The cracks and fissures caused by the addition of BSO in the film formulation could also be a reason for the increase in WVP. The interaction between the oil and the hydrophilic chain of the protein may also reduce the hydrophobicity of the film matrix, leading to an increase in its WVP (Hosseini et al., 2015). Therefore, it is logical that adding a hydrophobic compound to the film formulation would reduce the WVP of the films. The results are consistent with the results of Hosseini et al. (2015) that with increasing the oregano EO, the WVP of gelatin‐chitosan‐based films increased.

#### 3.2.3 Color and opacity properties

The color properties of food packaging films play an important role in their appearance and consumer acceptance (Almasi et al., 2020). Table 1 presents the color properties of films. As shown, by increasing the BSO concentration, the L* and a* decreased while the b* and ΔE increased in all film samples (*p* < .05). In other words, the amount of yellowness, greenness, and opacity of the films increased with increasing the amount of BSO. The lowest ∆*E* value and the highest L* were observed in control films, followed by WPEO5% ones, which could be attributed to the WPI covering of the oil. Based on the results, the control and WPEO, FO, and LNEO films exhibited the least Δ*E* and the highest lightness values at the same level of BSO, respectively. These changes are expected due to the inherent color of BSO, WPI, and lecithin. Edible films are generally preferred to be colorless and similar to synthetic polymers. This aligns with the results of Haghju et al. (2016), who found that chitosan films became darker with an increasing concentration of nettle extract‐loaded nanoliposomes. As shown in Table 2, in the ultraviolet range (360–400 nm), light transmission through the films is lower than in the visible light range (400–800 nm), and light transmittance increases with longer wavelengths. The addition of BSO to the pure SPI film resulted in reduced transparency (*p* < .05). It is not unexpected due to the dark green color and high opacity of BSO and lecithin. However, the results showed that all the film samples had very high transparency indicating a good dispersion of LNEO and WPEO within the SPI film matrix as observed by FE‐SEM images, which is comparable to synthetic films. According to the literature review, researchers found that the incorporation of EOs into the films yielded lower transparency values than those of the pure films, giving more opaque films (Binsi et al., 2013; Song et al., 2018). This phenomenon is probably because of an increase in light scattering induced by oil droplets in the film network.

**TABLE 2:** Light transmission and transparency of the SPI films.

Film sample	360	400	500	600	700	800	Transparency (%)
Control	62.23	71.77	74.47	75.85	76.03	79.06	79.76 ± 2.91^a^
FO5%	59.15	67.92	68.7	69.02	70.95	72.61	71.00 ± 2.91^c^
FO10%	66.22	70.14	72.44	76.38	78.88	79.61	54.71 ± 1.93^e^
LNEO5%	57.01	65.46	71.28	75.16	76.91	78.34	72.07 ± 2.45^c^
LNEO10%	58.88	66.68	70.79	73.79	76.38	79.98	67.01 ± 2.24^d^
WPEO5%	57.8	60.67	67.61	74.3	75.85	77.44	73.77 ± 0.64^b^
WPEO10%	48.97	55.59	61.8	62.66	66.06	69.5	71.48 ± 0.68^c^

*Note*: Data are the means of three replicates. Means with different letters within a column indicate significant differences (*p* < .05).

Abbreviations: Control, pure soy protein isolate; FO, free oil; LNEO, black seed oil‐loaded nanoemulsion in lecithin; WPEO, Pickering emulsion in whey protein isolate.

#### 3.2.4 Mechanical properties

Data on the UTS and STB of the films are presented in Table 1. The UTS and STB values for the control film were 7.6 ± 0.1 N m^−2^ and 33.56 ± 0.07%, respectively. The incorporation and increase in BSO levels, such as FO, LNEO, and WPEO, led to a significant (*p* < .05) decrease in the UTS values of the film samples. Excessive lipid addition can reduce film network cohesion, leading to the rupture of the composite film. This is because unsaturated fatty acids have a low ability to produce continuous films at high concentrations. The softening ability of lipids leads to a reduction in the tensile strength of edible films by attenuating intermolecular forces between adjacent chains. This also had been investigated by other researchers. For example, the chitosan/ascorbic film had a UTS value of 299 kgf cm^−2^ which then decreased to 151 kgf cm^−2^ when incorporated with lemongrass EO (Imawan et al., 2022). Although the addition of BSO by 5% increased the UTS, a further increase in oil (10%) led to a decrease in the UTS of the films. This may be because oil molecules have a plasticizing effect that reduces the molecular strength of the film by weakening the intermolecular connections in the protein chains. On the other hand, due to the reduced molecular density, flexibility decreased in higher concentrations of BSO (Almasi et al., 2020). Hence, the presence of oil reduces the mechanical strength of the SPI film. This finding aligns with a study by Ghadetaj et al. (2018) on WPI films, in which the tensile strength increased with the addition of 0.5% Grammosciadium pterocarpum Bioss, but NE decreased when 1% was added. Previous studies have also shown that the addition of NE can reduce UTS due to the plasticizing effect of NE droplets, which weakens intermolecular interactions between polymeric chains, improving film extensibility (Almasi et al., 2020; Pérez‐Córdoba et al., 2018), consistent with the current research.

The STB value of pure SPI films (33.56 ± 0.07) decreased with the incorporation of BSO as LNEO (31.14 ± 0.12%) and FO (18.27 ± 0.08%), while it increased in WPEO films (54.56 ± 0.11%) (*p* < .05). The STB of films is affected by the mobility of the polymer chains. Oils and other emollients increase molecular mobility. The WPEO film had the highest STB in comparison to other studied films. Also, a significant increase in STB was observed by increasing the oil concentration in all SPI film samples which was in line with Haghju et al.'s (2016) results. Lipid molecules may be located between protein chains, reducing protein–protein interactions, and may also cause protein chains to slide together. The lowest STB was observed in FO samples, which could be due to a decrease in film network cohesion by free BSO addition. However, Vargas et al. (2009) stated that the reason for the decrease in the amount of elasticity of the oleic acid‐loaded chitosan film is the connections between the fatty substance and the film matrix.

The density of SPI films is shown in Table 1. As can be seen, by increasing the BSO, the density of the films decreases. Jafarzadeh et al. (2017) showed that the decrease in density may be due to an increase in thickness and consequently, an increase in volume, which is associated with an increase in oil content. Also, Gahruie et al. (2017) studied basil seed gum‐based films incorporated with *Zatariamultiflora* EO nanoemulsion and reported that EO addition to the film matrix decreases the film density. As the oil percentage increased in WPEO films, the density of the films also increased. This may be because of the superior interaction between the encapsulated oil and the film matrix, which enhances the density of the films. The FE‐SEM images also support these findings.

##### Antioxidant activity

The antioxidant activity of oils is usually due to their phenolic compounds. The antioxidant properties of films containing BSO are related to folic acids, triterpenes, monoterpene glycosides, and flavonoids (Makouie et al., 2021). As shown in Table 1, the WPEO films exhibited a higher antioxidant half‐life than FO films, which indicates WPIs' greater protection of BSO in comparison to FO‐loaded films over time. Also, WPEO films had a higher antioxidant half‐life than LNEO films, which could be due to a thicker wall and better protection against oxidation. Hemmatkhah et al. (2020) reported that the active film containing cumin EO, prepared by PE of WPI, and inulin, had higher antioxidant activity and higher release rate than the EO encapsulated in Tween 80 stabilized nanoemulsion.

#### 3.2.5 Morphological properties

**FIGURE 3:**
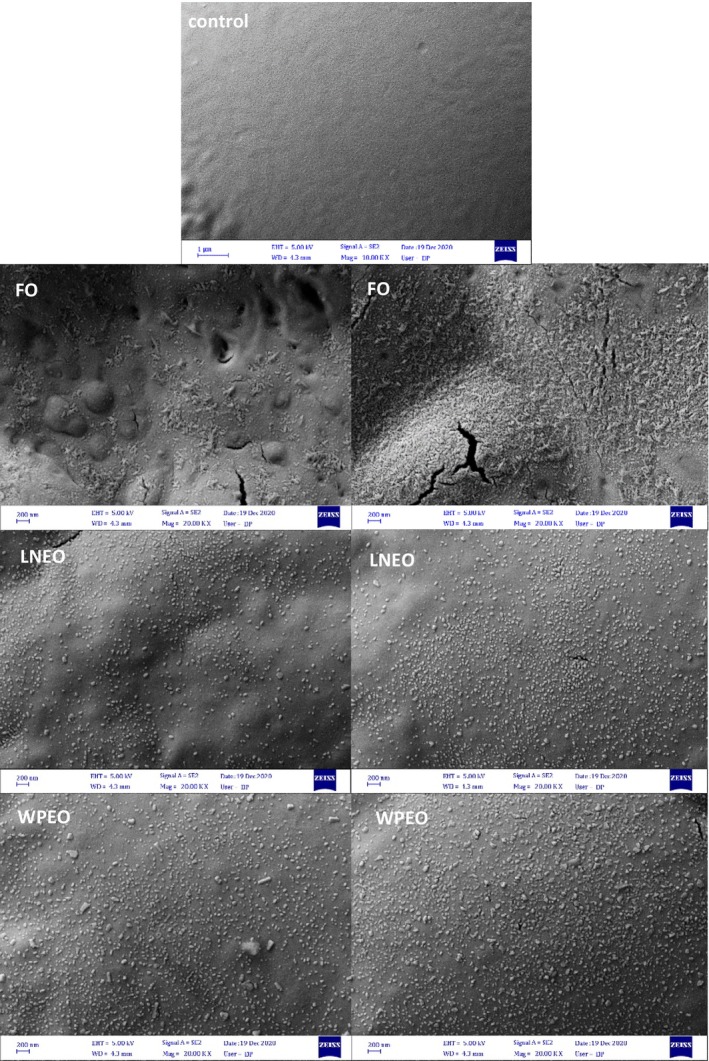
FE‐SEM images of the surface of control films and free or encapsulated BSO‐loaded films.

FE‐SEM images of the control film's surface and films containing free and encapsulated BSO are given in Figure 3. As shown, the control film has a dense, compact, and smooth surface and generally a homogenous surface without impurities. The surface roughness increased in the FO films, as expected for hydrophobic film samples. This is attributed to the lower capability of BSO to blend with the protein matrix. To confirm these results, particulates and large pores were observed for the 3% w/w cinnamon bark oil treatments due to improper interaction between the oil and the film matrix and the lack of uniform emulsion, observed clots, oil droplets, and cavities in the films (Ma et al., 2015). However, the LNEO samples showed a bubble‐like structure which may be attributed to the presence of NE droplets within the film matrix. Also, it has been reported that the holey structure of LNEO films may be due to the migration of oil droplets upward of the films and further volatilization during water evaporation (Almasi et al., 2020). FE‐SEM images of the surface of WEO films had a relatively smooth and dense surface compared to LNEO films and showed that the encapsulated oil was distributed better than FO in the film matrix. Interaction of wall materials with the SPI chains can fill in the gaps and thus give better compression to the structure. Also, it has been reported that in pectin‐based films, with the addition of MEO, the smooth and dense surface of the films was changed to an uneven structure (Almasi et al., 2020).

#### 3.2.6 FT‐IR spectroscopy

**FIGURE 4:**
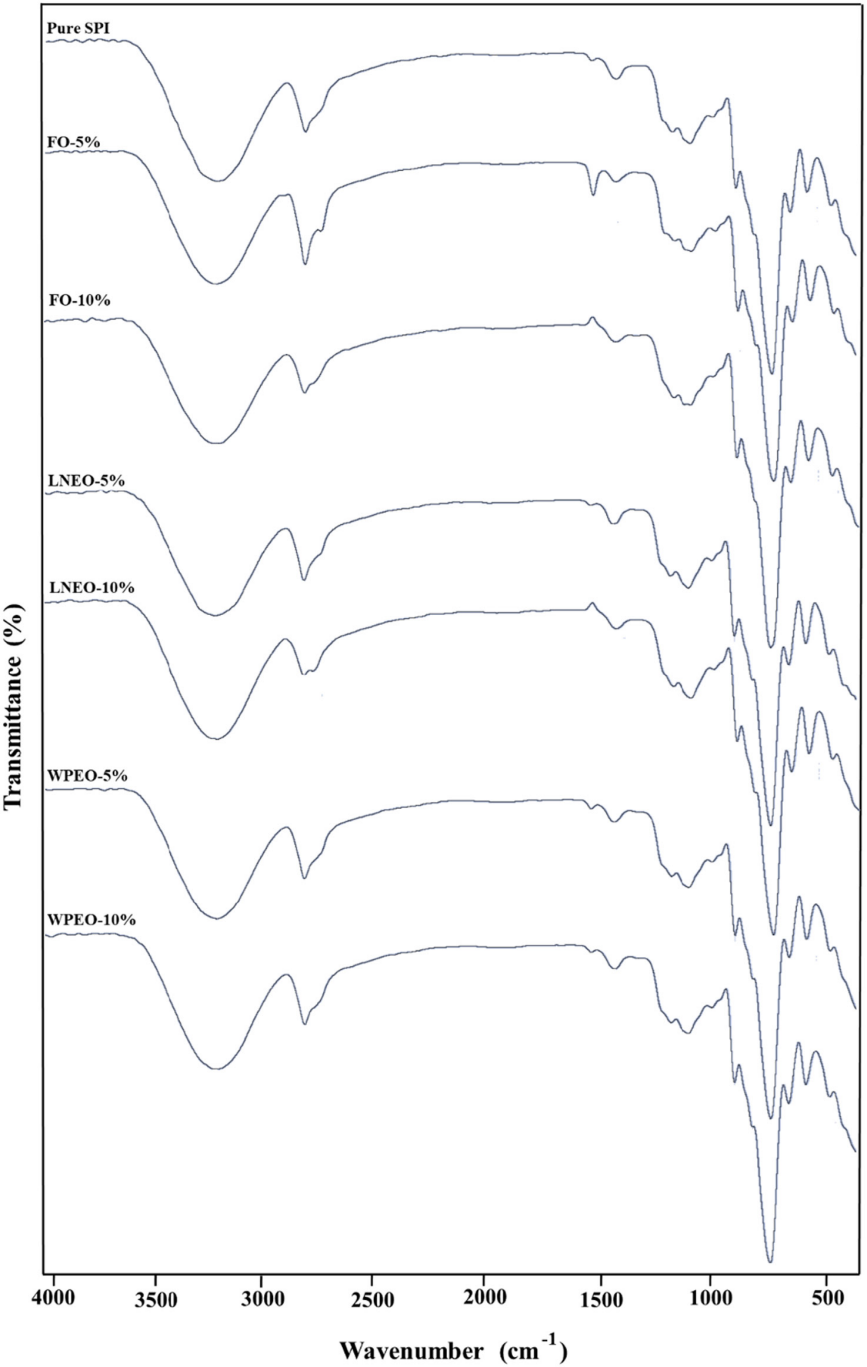
The FT‐IR spectra of film samples and pure SPI film.

To recognize the chemical structure and intramolecular interactions of the films, FT‐IR analysis was used. The FT‐IR spectra of the film samples are shown in Figure 4. The spectrum of the neat SPI film displayed distinct peaks at 288 cm^−1^, which indicates the presence of hydrogen bonds between water and SPI chains. Absorption peaks in the range of 2925 cm^−1^ belong to C–H stretching vibrations of the SPI chain. There is another distinct peak at 1545 cm^−1^ which belongs to the C=O stretching (amide I), and 1644 cm^−1^ was assigned to amide II, which corresponded to the C–N stretching and N‐H bending, respectively, and was selected to detect relative protein content (Wei et al., 2021). The absorption peak in the range of 1240–1413 cm^−1^ belongs to stretching bonds of N‐H bonds and C–N (Amide type III). Also, peaks in the range 1000 cm^−1^ are related to C–H bonds (Kalaycı et al., 2021), and the peaks around 756 cm^−1^ were attributed to stretching vibrations of C–C groups (Wu et al., 2018). The FT‐IR spectra of the control SPI film were almost identical to those of BSO‐loaded SPI films, and no change was observed in the functional groups. In general, all films showed a similar adsorption pattern, which means that the addition of BSO to the films did not cause a new peak in FT‐IR, and SPI film was stable in structure when BSO was added in free, NE, and PE forms. The same result was reported by Zhao et al. (2021) who studied the effect of the incorporation of different cotton‐nanocrystalline cellulose (C–NCC) contents into the SPI films. The films show similar IR peaks at 4000–500 cm^−1^, which indicates that the addition of C–NCC does not change the functional groups of the SPI films (Zhao et al., 2021). Also, Wang et al. showed that adding the anthocyanin‐rich red raspberry extract to the SPI films did not alter the major characteristics of the FT‐IR spectra of SPI film, therefore indicating no major alternations of the soy protein backbone (Wang et al., 2012).

#### 3.2.7 Biodegradability

The biodegradability evaluation shows the tendency of material compounds to be degraded by microorganisms. The biodegradability assessment of films determines their compatibility with the environment. The film samples showed a slight change in shape and texture after 21 days. No considerable change was observed in the shape and texture of the control film. The addition of BSO accelerated the degradation of films. At the highest percentage of BSO, the highest amount of degradation in the shape and texture of films was observed. The results are consistent with Medina Jaramillo et al. (2016), who stated that the addition of Yerba Mate extracts to cassava starch‐based films reduced the biodegradability time. The same results have been achieved by the addition of rosemary extract in cassava starch‐based films (Piñeros‐Hernandez et al., 2017) and polylactic acid to SPI films (González & Igarzabal, 2013). Overall, the excellent biodegradability of these developed SPI‐based films could offer a more suitable alternative to replace non‐degradable fossil fuels‐based packaging materials.

### 3.3 White bread quality attributes

#### 3.3.1 Shelf‐life extension of white bread

**FIGURE 5:**
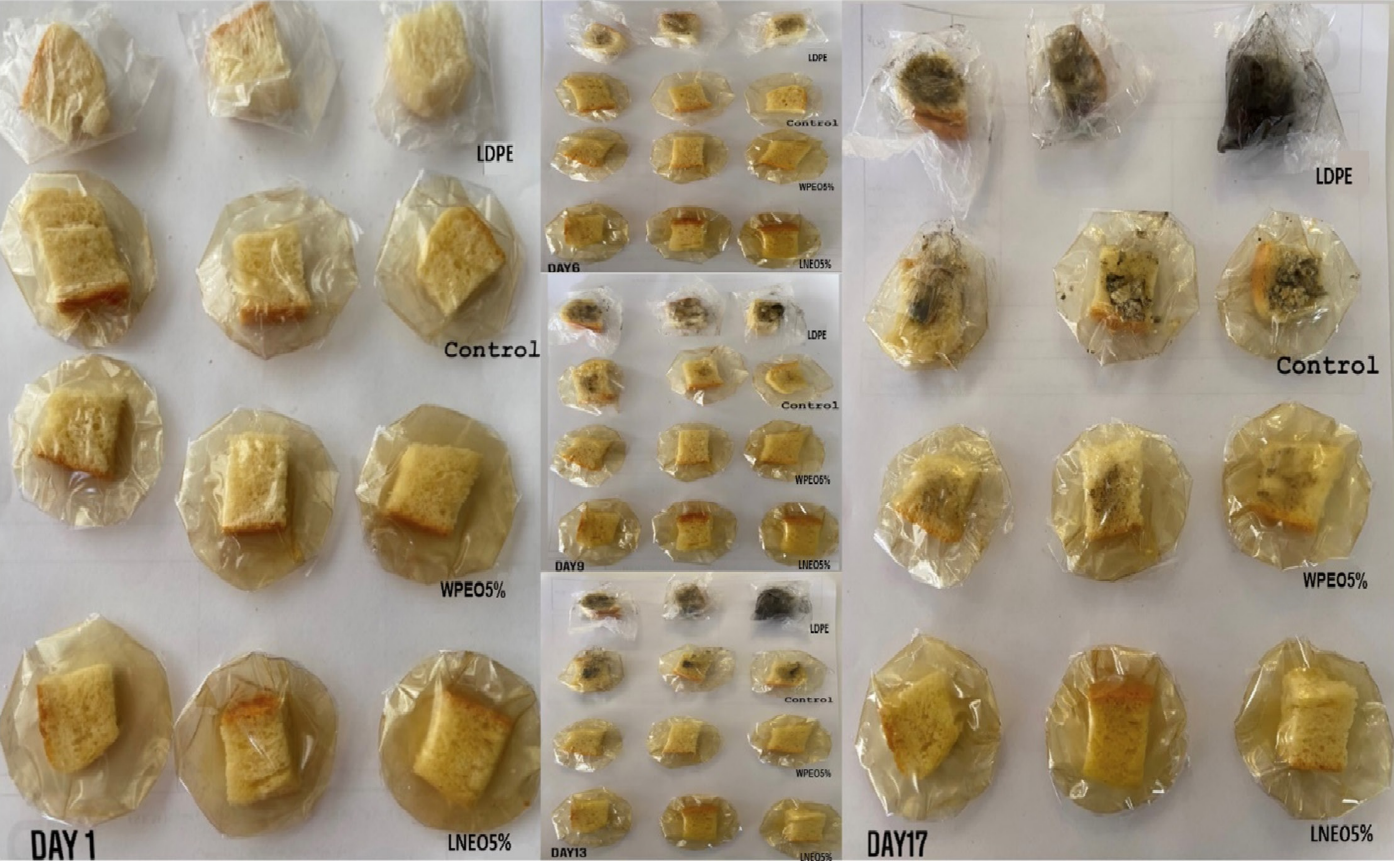
Visual appearance of white bread slices stored in LDPE, black seed oil‐loaded nanoemulsion in lecithin (LNEO), and Pickering emulsion in whey protein isolate (WPEO) and also pure SPI films (without oil) for 17 days.

Table 3 and Figure 5 display microbial growth and the visual appearance of white bread slices stored in pure SPI films (without BSO), as well as films incorporated with LNEO5%, WPEO5%, and LDPE (low‐density polyethylene) packages, for 17 days. No microbial growth was observed in LNEO‐containing samples until the end of the storage period. In WPEO‐containing packaging, the appearance of mold on white bread slices was observed on the 13th day of storage. Molds were observed on slices of bread packed with pure SPI film on the 9th day of storage. In the bread slices packed in the LDPE, mold was observed on the 6th day of storage, and the growth of mold in these samples proceeded rapidly. This underscores that the active film has a strong antifungal ability, which was also demonstrated in sensory assays. However, the mechanisms are not yet fully clarified and seem to be dependent on several factors, in particular, pH and moisture (Viscusi et al., 2021). Other literature has also indicated the shelf‐life extension of bread with active packaging. In white bread slices packaged in films produced from poly (butylene succinate)/geraniol and ethylene‐vinyl alcohol coating, no fungus was found on the white bread surface stored with an antimicrobial sachet for all 63 days of test duration, while the control sample was found to be spoiled earlier at day 21 (Petchwattana et al., 2021). Cinnamon and clove EOs were also found to effectively inhibit the mold growth of bakery products. It was found that cinnamon could prolong the shelf‐life of green bean cake for around 9–10 days, while clove oil did at around 3–4 days (Ju et al., 2018). Also, with eugenol and citral EOs antimicrobial sachet, the shelf life of bread stored in low‐density polyethylene, polypropylene, and high‐density polyethylene packages increased from 5 to 10, 13, and 15 days, respectively (Ju et al., 2020).

**TABLE 3:** Microbial growth and shelf‐life extension of white bread slices.

Film sample	Day 1	Day 3	Day 6	Day 9	Day 13	Day 17
LDPE	−	−	+	+	+	+
Pure SPI	−	−	−	+	+	+
WPEO5%	−	−	−	−	+	+
LNEO5%	−	−	−	−	−	−

Abbreviations: LDPE, low‐density polyethylene; LNEO, black seed oil‐loaded nanoemulsion in lecithin; pure SPI, pure Soy protein isolated film; WPEO, black seed oil‐loaded Pickering emulsion in whey protein isolate.

#### 3.3.2 Organoleptic evaluation of white bread slices

Organoleptic properties were evaluated on bread slices packaged in an LDPE package, pure SPI film, and BSO‐containing SPI films (LEO‐5% and WEO‐5%) for 9 days by 15 evaluators, and the data were analyzed by two‐way ANOVA. The sensory scores in this study range between 1 and 5 depending on the packaging type and storage time (Table 4). The results of ANOVA (Tables 5 and 6) indicated that packaging type and storage time, and their interaction significantly affected sensory properties (*p* < .05). The results showed that the highest sensory properties of bread samples were obtained from LNEO5% (Table 4) during the storage time. In general, the score of the sensory properties, including taste, texture, odor, and appearance, as well as the overall acceptance of bread slices, decreased over time in all samples (Table 6) (*p* < .05). LNEO‐packed samples showed the highest scores compared to the others, which indicates the slow staleness of bread slices with the presence of LNEO (Table 4). The reason for the smaller changes in bread wrapped in LNEO‐containing packaging may be due to the thinner wall of this type of capsule and the faster release of BSO as a preservative compared to the other samples. Bread slices packed in WPEO and pure SPI films also showed acceptable scores (>3) up to the 6th day after baking, while samples wrapped in LDPE packaging had the lowest score during storage time.

**TABLE 4:** Organoleptic scores of packed bread slices.

Packaging type	Time (day)	Taste	Texture	Odor	Appearance	Overall preference
LDPE	1	4.66 ± 0.577	4.33 ± 0.578	5.00 ± 0.000	4.67 ± 0.540	4.00 ± 0.000
	3	3.33 ± 0.577	3.00 ± 0.000	3.33 ± 0.578	4.00 ± 0.000	3.00 ± 0.000
	6	1.00 ± 0.000	1.66 ± 0.578	1.00 ± 0.000	1.00 ± 0.000	1.00 ± 0.000
	9	1.00 ± 0.000	1.00 ± 0.000	1.00 ± 0.000	1.00 ± 0.000	1.00 ± 0.000
Pure SPI	1	5.00 ± 0.000	5.00 ± 0.000	5.00 ± 0.000	5.00 ± 0.000	5.00 ± 0.000
	3	4.20 ± 0.000	3.33 ± 0.578	4.66 ± 0.576	4.33 ± 0.577	3.67 ± 0.567
	6	2.00 ± 0.200	2.33 ± 0.577	1.00 ± 0.000	3.00 ± 0.000	3.00 ± 0.000
	9	1.00 ± 0.000	1.33 ± 0.578	1.00 ± 0.000	1.00 ± 0.000	1.00 ± 0.000
LNEO5%	1	4.90 ± 0.330	5.00 ± 0.000	5.00 ± 0.000	5.00 ± 0.000	5.00 ± 0.000
	3	4.66 ± 0.577	4.67 ± 0.578	4.66 ± 0.576	5.00 ± 0.000	5.00 ± 0.000
	6	4.66 ± 0.577	4.00 ± 0.000	3.667 ± 0.577	4.33 ± 0.577	4.00 ± 0.000
	9	4.00 ± 0.000	3.33 ± 0.578	3.67 ± 0.577	4.00 ± 0.000	3.67 ± 0.567
WPEO5%	1	4.33 ± 0.577	4.67 ± 0.577	4.00 ± 0.000	5.00 ± 0.000	4.66 ± 0.576
	3	4.00 ± 0.00	4.00 ± 0.000	3.00 ± 0.000	4.33 ± 0.577	4.00 ± 0.000
	6	3.67 ± 0.567	3.67 ± 0.578	1.33 ± 0.577	4.00 ± 0.000	3.33 ± 0.577
	9	2.33 ± 0.577	2.00 ± 0.000	1.00 ± 0.000	1.67 ± 0.578	1.00 ± 0.000

*Note*: Values were mean ± SD.

Abbreviations: LDPE, low‐density polyethylene; LPEO, black seed oil‐loaded nanoemulsion in lecithin; Pure SPI, pure Soy protein isolated film; WPEO, black seed oil‐loaded Pickering emulsion in whey protein isolate.

**TABLE 5:** ANOVA of packaging type and storage time on color parameters.

Variable	Source	Mean square	*F*	Significant
Taste	Packaging type	9.611	65.905	0.00
	Time	16.222	111.238	0.00
	Packaging type × Time	2.167	14.857	0.00
Texture	Packaging type	6.833	36.440	0.00
	Time	17.44	93.040	0.00
	Packaging type × Time	0.648	3.457	0.00
Odor	Packaging type	8.743	69.944	0.00
	Time	28.965	231.720	0.00
	Packaging type × Time	1.54	12.310	0.00
Appearance	Packaging type	8.354	80.200	0.00
	Time	21.132	202.867	0.00
	Packaging type × Time	1.780	17.089	0.00
Overall preference	Packaging type	9.61	115.33	0.00
	Time	17.500	210.00	0.00
	Packaging type × Time	0.963	11.56	0.00

**TABLE 6:** Main factor analysis using Duncan's multiple range test of organoleptic of packed bread samples during storage.

	Taste	Texture	Odor	Appearance	Overall preference
Day					
1	4.75 ± 0.45^A^	4.75 ± 0.45^A^	4.75 ± 0.45^A^	4.83 ± 0.38^A^	4.66 ± 0.49^A^
3	3.91 ± 0.66^B^	3.75 ± 0.75^B^	3.91 ± 0.9^B^	4.41 ± 0.51^B^	3.91 ± 0.79^B^
6	2.91 ± 1.56^C^	2.91 ± 1.08^C^	1.75 ± 1.08^C^	3.08 ± 1.37^C^	2.83 ± 1.19^C^
9	2.08 ± 1.31^D^	1.91 ± 0.99^D^	1.66 ± 1.23^C^	1.91 ± 1.31^D^	1.91 ± 1.16^D^
Film samples					
LDPE	2.5 ± 1.67^d^	2.5 ± 1.38^d^	2.58 ± 1.78^c^	2.58 ± 1.67^d^	2.25 ± 1.35^d^
Pure SPI	3 ± 1.65^c^	3 ± 1.47^c^	2.91 ± 2.02^b^	3.33 ± 1.61^c^	3.16 ± 1.52^c^
LNEO5%	4.58 ± 0.51^a^	4.25 ± 0.75^a^	4.25 ± 0.75^a^	4.58 ± 0.51^a^	4.41 ± 0.66^a^
WPEO5%	3.58 ± 0.90^b^	3.58 ± 1.08^b^	2.33 ± 1.30^c^	3.75 ± 1.35^b^	3.5 ± 1.08^b^

*Note*: Values were mean ± SD. Means with the different superscript letters within a column indicate significant differences (*p* < .05).

Abbreviations: LDPE, low‐density polyethylene; LPEO, black seed oil‐loaded nanoemulsion in lecithin; pure SPI, pure Soy protein isolated film; WPEO, black seed oil‐loaded Pickering emulsion in whey protein isolate.

#### 3.3.3 Color properties

Color and appearance have an important effect on the acceptance of consumers. The values of color parameters in this study varied depending on the packaging type and storage time (Table 7). The results of ANOVA (Table 2) indicated that packaging type and storage time and their interaction significantly affected the color parameters of packed samples (*p* < .05). As shown in Tables 7 and 8, the L*, a*, and b* values of the samples showed a significant decrease during the storage time. The LDPE packaging sample showed the largest decrease in the L*, a*, and b* values, and the LNEO‐containing sample showed the smallest change between samples. Also, the ∆*E* value of bread slices significantly increased during 17‐day storage. Bread slices packed in SPI films containing BSO (LNEO5%, WPEO5%) showed the least ∆*E*, while the LDPE‐packed sample showed the highest ∆E, followed by a sample wrapped in Pure SPI film (Tables 7 and 9). These observations show the effect of BSO on the preservation of color properties in bread and may be attributed to the antioxidant and antimicrobial activity of bioactive compounds presented in BSO. In general, the sample packed with LNEO‐containing film showed the best results in comparison to other kinds of packaging in terms of the color characteristics of the bread.

**TABLE 7:** Color parameters of packed bread slices.

Packaging type	Time (day)	L*	a*	b*	Δ*E*
LDPE	1	53.71 ± 0.026	29.70 ± 0.074	43.85 ± 0.036	66.84 ± 0.040
	6	33.67 ± 0.045	17.69 ± 0.020	31.43 ± 0.015	71.92 ± 0.030
	17	9.01 ± 0.020	6.62 ± 0.030	10.93 ± 0.015	89.25 ± 0.019
Pure SPI	1	51.74 ± 0.032	30.66 ± 0.020	41.55 ± 0.030	67.44 ± 0.019
	6	42.78 ± 0.036	23.94 ± 0.030	36.57 ± 0.020	68.51 ± 0.024
	17	13.89 ± 0.015	11.51 ± 0.020	17.05 ± 0.025	85.73 ± 0.009
LNEO5%	1	52.53 ± 0.020	30.12 ± 0.010	41.45 ± 0.010	66.57 ± 0.021
	6	51.87 ± 0.020	29.78 ± 0.026	40.87 ± 0.015	66.54 ± 0.008
	17	48.94 ± 0.015	27.96 ± 0.025	39.24 ± 0.020	66.87 ± 0.030
WPEO5%	1	53.00 ± 0.010	30.89 ± 0.015	42.11 ± 0.020	67.00 ± 0.013
	6	49.15 ± 0.030	25.98 ± 0.010	38.06 ± 0.035	65.20 ± 0.010
	17	35.66 ± 0.030	13.35 ± 0.025	28.74 ± 0.025	67.96 ± 0.013

*Note*: Values were mean ± SD. * used to show the color parameters.

Abbreviations: LDPE, low‐density polyethylene; LPEO, black seed oil‐loaded nanoemulsion in lecithin; Pure SPI, pure Soy protein isolated film; WPEO, black seed oil‐loaded Pickering emulsion in whey protein isolate.

**TABLE 8:** ANOVA of packaging type and storage time on color parameters.

Variable	Source	Mean square	*F*	Significant
L*	Packaging type	685.70	94,2181.04	0.00
	Time	2090.73	2,872,762.126	0.00
	Packaging type × Time	282.28	387,861.84	0.00
a*	Packaging type	196.37	216,855.59	0.00
	Time	731.40	807,685.63	0.00
	Packaging type × Time	67.065	74.059.11	0.00
b*	Packaging type	240.92	429,370.32	0.03
	Time	1051.69	1,874,301.00	0.00
	Packaging type × Time	141.86	252,819.54	0.00
Δ*E*	Packaging type	211.17	430,919.38	0.00
	Time	399.73	815,674.84	0.00
	Packaging type × Time	112.30	229,153.94	0.00

*Note*: * used to show the color parameters.

**TABLE 9:** Main factor analysis using Duncan's multiple range test for color properties of packed bread samples during storage.

	L*	a*	b*	△*E*
Day				
1	52.74 ± 0.74^A^	30.34 ± 0.48^A^	42.24 ± 1^A^	66.96 ± 0.33^C^
6	44.36 ± 7.31^B^	24.34 ± 4.57^B^	36.73 ± 3.58^B^	77.04 ± 2.64^B^
17	26.87 ± 16.93^C^	14.85 ± 8.30^C^	23.98 ± 11.36^C^	78.45 ± 10.57^A^
Film samples				
LDPE	32.12 ± 19.39^d^	18 ± 9.99^d^	28.73 ± 14.39^d^	76.45 ± 10.17^a^
Pure SPI	36.13 ± 17.13^c^	22.03 ± 8.41^c^	31.72 ± 11.21^c^	73.89 ± 8.89^b^
LNEO5%	51.11 ± 1.65^a^	29.28 ± 1.00^a^	40.52 ± 0.99^a^	66.65 ± 0.15^d^

*Note*: Values were mean ± SD. The means that the different superscript letters within a column show significant differences (*p* < .05). * used to show the color parameters.

Abbreviations: LDPE, low‐density polyethylene; LNEO, black seed oil‐loaded nanoemulsion in lecithin; Pure SPI, pure Soy protein isolated film; WPEO, black seed oil‐loaded Pickering emulsion in whey protein isolate.

## 4 CONCLUSION

Bread is a widely consumed product worldwide, but it is susceptible to rapid fungal decay, leading to the production of toxins and economic losses. The quality and safety properties of bakery products are notably depending on packaging materials and technologies. To address this issue, biodegradable active packaging has been proposed as a solution. It not only enhances consumer acceptability but also offers a safe way to inhibit the growth and multiplication of various microorganisms in bakery products. According to this fact, this study is based on incorporating the BSO in the forms of LNEO, WPEO, and free with SPI as a new composite film. This incorporation affected the color, antioxidant, antimicrobial, WVP, microstructure, physical, and mechanical properties of the SPI film samples. The edible films containing LNEO5% and WPEO5% were then chosen for use in the packing of white bread slices based on the physical and mechanical results. The shelf‐life extension study informed that the corruption of bread wrapped with LNEO5% films was delayed by more than 17 days, while it was 6 and 9 days for ones packed in LDPE and pure SPI, respectively. In conclusion, this study suggests the application of SPI films activated by BSO‐loaded PE and NE as new active packaging, showing great promise in extending the shelf‐life of bread and reducing waste.

## AUTHOR CONTRIBUTIONS

**Negin Hoseinieh:** Investigation (equal); methodology (equal). **Forogh Mohtarami:** Conceptualization (equal); data curation (equal); formal analysis (equal); software (equal); supervision (equal); validation (equal); writing – review and editing (equal). **Hadi Almasi:** Conceptualization (equal); data curation (equal); supervision (equal); review and editing (equal). **Saeedeh Azizi:** Writing – original draft (equal).

## CONFLICT OF INTEREST STATEMENT

The authors declare no competing interests.

## Data Availability

Data will be made available on reasonable request.
